# Complement Inhibitors from Scabies Mites Promote Streptococcal Growth – A Novel Mechanism in Infected Epidermis?

**DOI:** 10.1371/journal.pntd.0001563

**Published:** 2012-07-17

**Authors:** Angela Mika, Simone L. Reynolds, Darren Pickering, David McMillan, Kadaba S. Sriprakash, David J. Kemp, Katja Fischer

**Affiliations:** 1 Infectious Diseases Program, Biology Department, Queensland Institute of Medical Research, Herston, Brisbane, Australia; 2 School of Veterinary Sciences, The University of Queensland, Gatton, Australia; George Washington University, United States of America

## Abstract

**Background:**

Scabies is highly prevalent in socially disadvantaged communities such as indigenous populations and in developing countries. Generalized itching causes discomfort to the patient; however, serious complications can occur as a result of secondary bacterial pyoderma, commonly caused by *Streptococcus pyogenes* (GAS) or *Staphylococcus aureus*. In the tropics, skin damage due to scabies mite infestations has been postulated to be an important link in the pathogenesis of disease associated with acute rheumatic fever and heart disease, poststreptococcal glomerulonephritis and systemic sepsis. Treatment of scabies decreases the prevalence of infections by bacteria. This study aims to identify the molecular mechanisms underlying the link between scabies and GAS infections.

**Methodology/Principal Findings:**

GAS bacteria were pre-incubated with blood containing active complement, phagocytes and antibodies against the bacteria, and subsequently tested for viability by plate counts. Initial experiments were done with serum from an individual previously exposed to GAS with naturally acquired anti-GAS antibodies. The protocol was optimized for large-scale testing of low-opsonic whole blood from non-exposed human donors by supplementing with a standard dose of heat inactivated human sera previously exposed to GAS. This allowed an extension of the dataset to two additional donors and four proteins tested at a range of concentrations. Shown first is the effect of scabies mite complement inhibitors on human complement using ELISA-based complement activation assays. Six purified recombinant mite proteins tested at a concentration of 50 µg/ml blocked all three complement activation pathways. Further we demonstrate in human whole blood assays that each of four scabies mite complement inhibitors tested increased GAS survival rates by 2–15 fold.

**Conclusions/Significance:**

We propose that local complement inhibition plays an important role in the development of pyoderma in scabies infested skin. This molecular link between scabies and bacterial infections may provide new avenues to develop alternative treatment options against this neglected disease.

## Author Summary

Australian Aborigines experience streptococcal invasive diseases at a five times greater rate than the general Australian population [1], contributing to an estimated life expectancy gap of currently 13 years [2] with infectious diseases being the major cause in remote areas. Alternative approaches to control skin infection and associated complications in Aboriginal communities are imperative [3]. A clear link between scabies and bacterial pyoderma has been recognised as an underlying factor of Aboriginal cases of rheumatic fever and heart disease (RF/RHD), skin sepsis and renal disease [4]. Aiming to elucidate the causal molecular mechanisms we identified multiple scabies intestinal protein families functioning as inhibitors of human complement pathways, thereby preventing complement-mediated gut damage. The work presented here is at the forefront of a new agenda, looking at the interactions between scabies mites, bacteria and the host. We show that scabies mite proteins act as complement inhibitors and enhance GAS growth in whole blood assays, presumably by inhibition of host innate immunity. Based on this groundwork data we hypothesize that the complement-inhibitory functions of excreted gut molecules promote the growth of bacterial pathogens in the microenvironment of the epidermal burrows.

## Introduction

The global prevalence of pyoderma from various bacterial infections has been estimated to exceed 111 million children, making it one of the most common skin afflictions along with scabies and tinea [5]. In tropical climates, scabies predisposes to secondary bacterial skin infections in particular by *Streptococcus pyogenes* (group A streptococci, GAS), the causal agent of acute rheumatic fever and rheumatic heart disease (ARF/RHD). This association between scabies and pyoderma caused by GAS has been well established [4]. Globally, GAS associated diseases, such as RHD, acute post-streptococcus glomerulonephritis (APSGN) and severe invasive diseases, affect an estimated 18 million individuals and account for over 0.5 million deaths per year [6]. In Australian Aboriginal communities RF/RHD prevalence has steadily risen to almost 2% in 2008 [7], translating to the highest incidences reported globally. Scabies and pyoderma have also been linked with outbreaks of APSGN [4]. In remote Aboriginal communities more than 70% of the children below two years of age have presented with scabies and skin sores, respectively [8]. Community-wide treatment of scabies decreases pyoderma [9], [10], which suggests a key role of the burrowing mite.

Mechanical disruption of the stratum corneum caused by mites and host scratching may be considered a primary prerequisite promoting secondary skin infections; however contributing molecular interactions between host, parasites and bacteria have not been investigated. An immediate, non-specific epidermal host defense mechanism against microbes is the local activation of the complement system [11]. Phagocytes migrate to the site of infection and attempt to engulf and dispose of the invading organisms with the help of available antibodies and complement, both present in the host's serum, [12]. We have recently established that scabies mites express extensive complement evasion machinery disrupting the three complement pathways at several levels. Two members of a large family of catalytically inactive serine protease paralogues termed SMIPP-Ss [13], [14] inhibit all three pathways of human complement [15]. Furthermore two scabies mite serpins (SMSs) also function as effective complement inhibitors, each binding to a range of complement factors on several levels of the three complement pathways [16]. While the *in vivo* concentrations of these mite complement inhibitors excreted into the confined space of the burrows have not been determined, cumulative effects of multiple anti-complement activities can be expected. A logical question to ask is whether this increased level of anti-complement activity has an effect on the bacteria that colonize the burrows. Here we present a set of *in vitro* data focusing on two SMIPP-Ss and two SMSs, which indicate that under physiological conditions there is indeed a considerable effect on the growth of *Streptococcus pyogenes*.

## Methods

### Ethics statement

Normal human serum for complement activation assays and whole blood samples for bactericidal assays were prepared from blood donated by healthy volunteers after informed consent and in accordance with the principles in ethical conduct as stated in the “National Statement on Ethical Conduct in Human Research”, documented by the Australian National Health and Medical Research Council.

### Cloning

Five scabies mite inactive serine protease paralogues (*SMIPP-S D1*, GenBank accession no. AY333085, *SMIPP-S I1*, AY333081; *SMIPP-S B2*, AY333073; *SMIPP-S G2*, JN167504 and *SMIPP-S G4*, AY333078;) were cloned into *Pichia pastoris* KM71H using vector pPICZαA (Invitrogen) as described earlier [15]. Two scabies mite serpins (SMSs; *SMSB3a*, cDNA clone Yv7088B02; GenBank accession no. JF317220; *SMSB4*, cDNA clone Yv5004A04, GenBank accession no. JF317222) were amplified on cDNA libraries made from human scabies mites *Sarcoptes scabiei* [17], [18] and cloned into the pQE9 expression vector (Qiagen) [16].

### Heterologous expression and purification of SMIPP-Ss and SMSs

Recombinant SMIPP-S proteins were expressed in *P. pastoris* as secreted proteins and purified as described earlier [15]. Briefly, mature SMIPP-S protein secreted from *P. pastoris* was purified from the expression culture supernatant by hydrophobic interaction chromatography on a 5 ml HiTrap phenyl-Sepharose column (GE Healthcare) followed by dialysis and ion chromatography on a 5 ml HiTrap SP Sepharose FF column (GE Healthcare). Recombinant SMS proteins were expressed in *E. coli* and purified under denaturing conditions from thoroughly washed and solubilised inclusion bodies by nickel immobilized metal affinity chromatography (Qiagen). Purified SMS proteins were refolded for 3 hours in 300 mM L- arginine, 50 mM Tris, 50 mM NaCl and 5 mM DTT at pH 8.0 for SMSB3 and pH 10.5 for SMSB4. Refolded SMS proteins were concentrated using an Ultrasette Lab Tangential Flow Device (10 kDa cut off; PALL Life Sciences), followed by further concentration in centrifugal filters (Amicon Ultra, Millipore).

Molecular masses and purity of the purified recombinant mite proteins were confirmed using SDS-PAGE analysis with silver and Coomassie blue R-250 staining. Protein concentrations were determined according to the Bradford method [19]. Prior to the phagocytosis assays, the recombinant mite proteins were buffer exchanged into the corresponding assay buffers using Zeba Desalt Spin columns (Pierce).

### Complement activation assays

Human serum complement levels were assessed using a Wieslab Complement System Screen kit (EuroDiagnostica) according to the manufacturer's instructions. Normal human serum was prepared from blood of eight healthy volunteers after informed consent. Inhibition of complement by five SMIPP-Ss and one SMS was measured in a total volume of 100 µl at serum concentrations of 1%, 1% and 5.5% for the classical, lectin and alternative complement pathways, respectively. These serum concentrations represent recommended conditions, under which each assay is most sensitive to changes. Normal human serum was pre-incubated for 30 min at room temperature with 50 µg/ml of purified scabies mite protein before addition to the ELISA microtiter plate and immunodetection of the terminal membrane attack complex (MAC, C5b-9). Absorbance was measured at a wavelength of 405 nm on a POLARstar Optima fluorescent microtiter plate reader (BMG Labtech, Melbourne, Australia). The absorbance obtained in the absence of SMIPP-Ss was defined as 100%.

### Bacterial strain

*S. pyogenes* (GAS strain 2967, emm-type emm 1) was originally isolated from a patient with APSGN in Townsville, Queensland, Australia.

### Bacterial growth in human whole blood

Informed consent was obtained from all blood donors. The initial set of phagocytosis assays was performed using blood from an individual previously exposed to GAS (D1) with a robust type-specific immune response to the GAS strain 2967.

The standardized phagocytosis assays used blood from “nonimmune” human donors who did not exhibit type-specific immunity to GAS 2967. Among several donors tested in preliminary experiments, blood from most donors allowed growth of the GAS strain, and blood from two such donors was used in each set of phagocytosis experiments (D2 and D3). To reduce differences in bacterial growth based on differences in anti-GAS antibody levels of individual whole blood donors, bactericidal assays were standardized for donors without or with low levels of naïve anti-GAS IgG by addition of antibodies from a donor previously exposed to GAS (D1). Immediately prior to use added antibody sera were heat-inactivated at 56°C for 15 min to abolish complement activity.

The initial set of bactericidal assays was performed with human whole blood collected in a standard BD vacutainer (Becton, Dickinson and Company), using sodium heparin as anticoagulant at a concentration of 15 USP Units/ml. Comparison of heparin- versus hirudin-treated blood in bactericidal assays confirmed earlier findings [20] that the anticoagulant heparin can alter complement activation, thereby affecting bacterial survival. Thus, further bactericidal assays were carried out using hirudin (lepirudin) as anticoagulant, at a concentration of 25 µg/ml (Dynabyte Informationssysteme GmbH, Munich, Germany).

The assays were performed as described previously [21] with modifications. Bacteria were grown overnight without agitation at 37°C in 5 ml Todd-Hewitt Broth (THB). A dilution of this pre-culture was grown in THB with agitation to early exponential growth phase (OD_600_ 0.1), and then diluted in PBS to 10^−2^ or 10^−3^, representing on average 6×10^3^ colony forming units (CFU) per ml. Per assay 100 µl human venous blood, 12.5 µl antibody serum, which was heat-inactivated for 15 min 56°C in a water bath, 15 µl of scabies mite protein as complement inhibitor or BSA as negative control (final concentration 25–400 µg/µl) in GVB^2+^ buffer (5 mM veronal, pH 7.35, 140 mM NaCl, 0.1% (w/v) gelatin, 1 mM MgCl_2_, 0.15 mM CaCl_2_) and 12.5 µl bacteria (containing on average approximately 75 CFU) were added to a total volume of 140 µl. Assays were placed on a rotisserie and incubated by end over end mixing for 3 h at 37°C. Subsequently 50 µl aliquots from each tube were plated in duplicate on 2.5% (v/v) defibrinated horseblood THB agar (Equicell, Australia) using the pour plate method and incubated overnight at 37°C for enumeration of CFU. Bacteria growth may vary between assays performed on different occasions and between different donors. Hence the percentage difference in bacterial growth was calculated by comparing CFU recovered after addition of scabies mite proteins against CFU recovered from buffer controls at the same time points in individual experiments. Individual assays were performed in duplicates and repeated independently between 4 and 12 times.

### Statistical analysis

Statistical significance was determined using t tests (GraphPad Prism software, version 5.0; GraphPad Software Inc. USA). Values of *p*<0.05 were considered significant.

## Results

### SMIPP-Ss and SMSs inhibit all three human complement activation pathways

**Figure 1:**
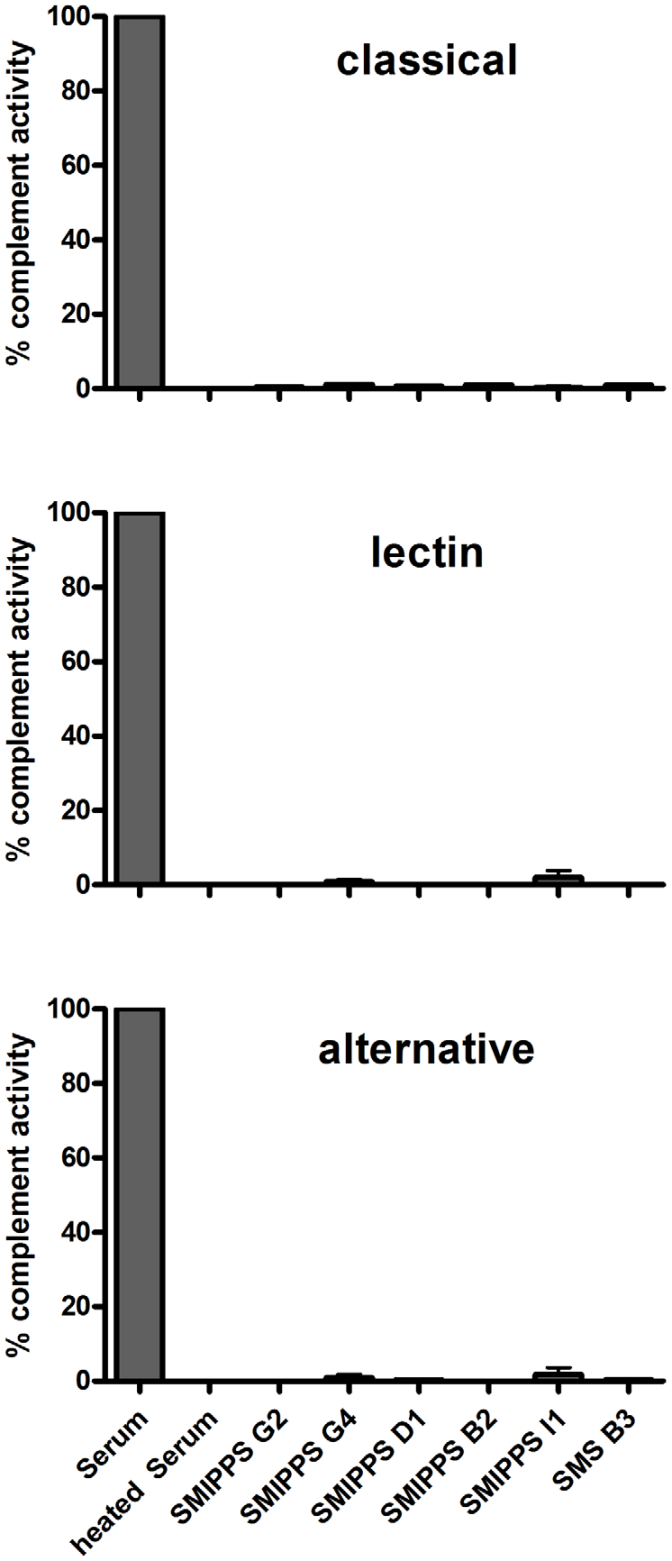
Inhibition of human complement by excretory mite proteins. Shown are the effects of five recombinant, purified SMIPP-Ss (I1, D1, B2, G2, G4) and one SMS (B3) on the Classical (CP), Lectin (LP) and Alternative (AP) pathways of the human complement system. In ELISA-based complement activation assays 50 µg/ml of each recombinant protein showed strong complement inhibition. Data presented were obtained in triplicates and is representative for three to five independent experiments for each mite protein. Shown is the percentage of complement activity as the mean ± SD.

A simple method comprising three ELISAs, originally developed to screen for complement deficiencies [22], was employed to confirm the complement-inhibitory properties of the recombinant mite molecules investigated here. The assays were performed with normal human serum and detection of activation was determined as the incorporation of C9 into the terminal membrane attack complex. In this system six purified recombinant mite proteins (five SMIPP-Ss and one SMS) at a final concentration of 50 µg/ml inhibited all three complement activation pathways (Figure 1).

### Standardization of whole blood bactericidal assays to test the effect of SMIPP-Ss and SMSs on bacterial survival

**Figure 2:**
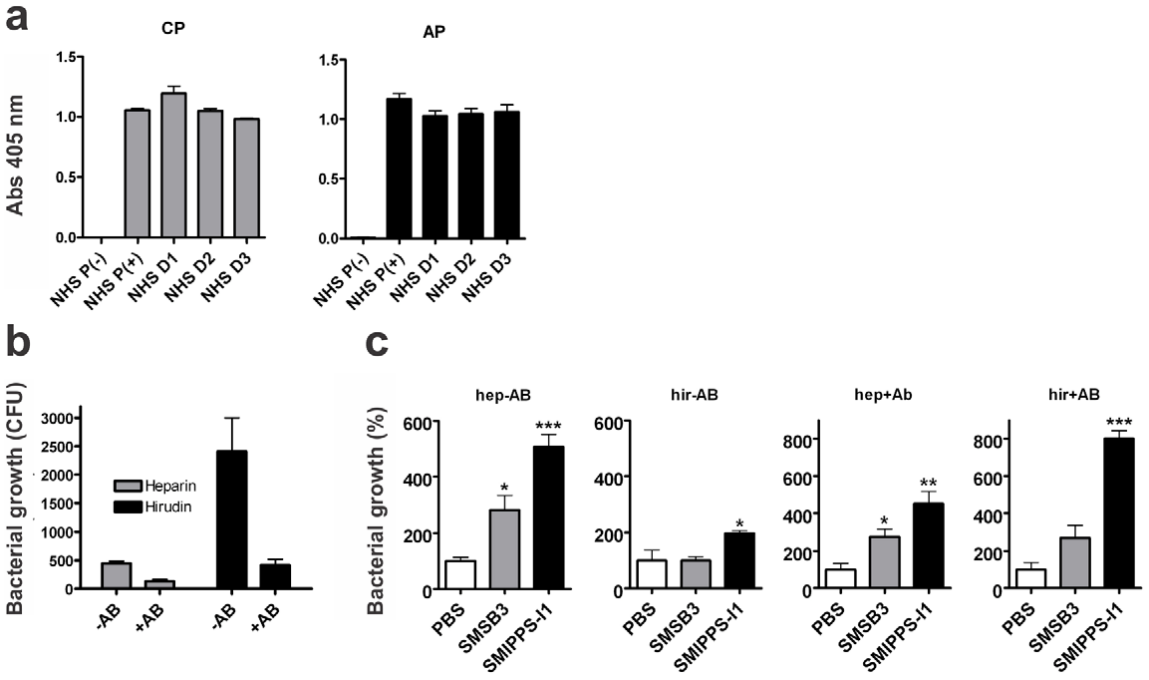
Assessment of donor blood and the role of anticoagulant and antibody levels in bactericidal assays. (**a**) The three human whole blood donors used in this study showed average complement activation levels representative of the normal population. Complement activation of the Classical (CP) and Alternative (AP) pathways was determined by ELISA. Shown are means ± SEM of n = 2 independent experiments, each performed in duplicate. NHS P(−), inactivated; NHS P(+), active normal human serum (n = 8 donors); D1–D3, individual active serum donors. (**b**) Effect of anticoagulant on human whole blood bactericidal assays. Comparison of heparin- and hirudin-treated blood in bactericidal assays indicates that the anticoagulant heparin can alter complement activation at low concentrations, thereby affecting GAS growth. ±AB, with and without addition of heat-inactivated anti-GAS sera containing type-specific antibodies; (**c**) Scabies mite proteins enhance bacterial growth in GAS bactericidal assays under all conditions tested. Protein concentrations of 200 µg/ml were used. Shown are means ± SEM of n = 4 experiments from one representative blood donor with low naïve levels of anti-GAS antibodies. ±AB, with and without addition of heat-inactivated anti-GAS sera; hep, heparin; hir, hirudin. *, p<0.05; **, p<0.01; ***, p<0.001 by t test (GraphPad Prism software).

Prior to phagocytosis assays samples from three individuals (D1, D2, D3) were assessed for activation of the classical and alternative pathways by ELISA for detection of the deposition of C5b-C9, *i.e.* the terminal complement membrane attack complex. The complement activation levels from the three donors were similar to those of pooled normal human serum (Figure 2a), thereby validating the suitability of the donors.

In whole blood bactericidal assays heparin treated blood samples resulted in recovery of fewer GAS colonies than samples from the same donor treated with hirudin (Figure 2b). This confirmed earlier findings that the anticoagulant heparin can alter complement activation, while hirudin (lepirudin) generally preserved the complement reactivity, making it more suited for *in-vitro* studies [20], [23]. Thus, further bactericidal assay analysis was carried out using hirudin.

We aimed to investigate the effect of mite complement inhibitors on bacterial growth in blood from several donors, however most individuals tested did not show a type-specific immune response to the GAS strain 2967 (data not shown). To determine whether blood from “non-immune” donors (*i.e.* blood that did not contain type-specific opsonizing antibodies) was suitable, assays were conducted with or without the addition of such antibodies. Assays testing one SMIPP-S and one SMS at a concentration of 200 µg/ml generally resulted in increased bacterial growth; however, the most striking effects of mite proteins were seen in the presence of strain specific antibodies. One exemplary set of these results is shown in Figure 2c.

### Testing the effect of SMIPP-Ss and SMSs on bacterial survival in whole blood assays

**Figure 3:**
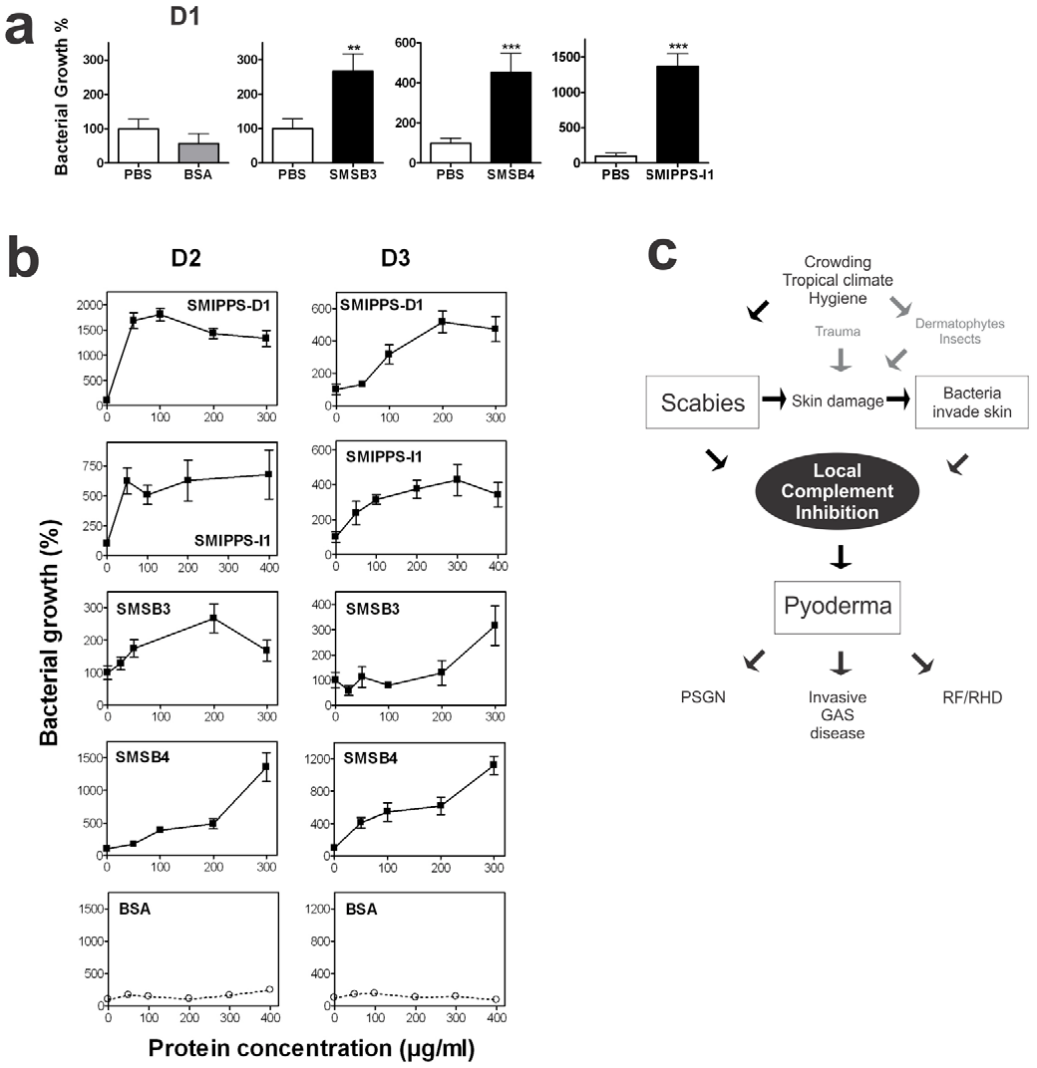
Scabies mite complement inhibitors enhance group A streptococcal growth in human whole blood. (**a**) Increased GAS growth in whole blood from a human donor with a type-specific immune response to the GAS strain investigated (D1) in presence of SMSB4 [25 µg/ml], SMSB3, SMIPP-S I1 or BSA [200 µg/ml]. Shown are means ± SEM (n = 4 experiments). (**b**) Means ± SEM of n = 6 to 12 standardized assays with whole blood from two donors who did not exhibit type-specific immunity to the GAS strain used in this study. (D2, D3) supplemented with heat-inactivated antibody serum from D1. (c) Novel model on potential scabies, bacteria and host interactions. **p<0.01, ***p<0.001 by t test (GraphPad Prism). *S. pyogenes* strain 2967emm1 was isolated from human skin.

Initial experiments were performed with plasma from an individual (D1) previously exposed to GAS and thus with a robust type-specific immune response to the GAS strain. A dramatic increase in the bacterial growth ranging from over 200 to almost 1500% was seen in the presence of SMSB3 (200 µg/ml), SMSB4 (25 µg/ml) and SMIPP-S I1 (200 µg/ml), compared to no effect of BSA (200 µg/ml), indicating that the mite proteins efficiently interfered with bacterial uptake by human phagocytes (Figure 3a).

As it was difficult to recruit further blood donors with high antibody titers, whole blood from non-exposed human donors was used and supplemented with a standard dose of heat-inactivated human sera previously exposed to GAS (D1). This allowed an extension of the dataset to two additional donors (D2 and D3) and four proteins, tested at a range of concentrations. Similarly to what was observed in the immune-competent donor, the presence of each scabies mite complement inhibitor increased bacterial survival rates considerably in a dose dependent manner (Figure 3b). The highest increases ranged from 200–300% for SMSB3, 400–600% for SMIPP-S I1 to over 1000% for SMSB4 and SMIPP-S D1, while bacterial growth was unchanged when the same amount of BSA bovine serum albumin was added instead.

## Discussion

We have previously demonstrated that two SMIPP-Ss are potent inhibitors of the human complement system, interfering with all three pathways of the complement cascade [15]. To assess the effect of additional mite molecules on complement, a microtiter plate-based deposition assay was performed in which complement activation was initiated by specific ligands for each pathway. After addition of human serum, pre-treated with the purified recombinant mite proteins, deposited complement proteins were detected using specific Abs against the terminal membrane attack complex (MAC, C5b-9). We showed that three further SMIPP-Ss from additional clades within the phylogenetic tree of the SMIPP-S family [14] and one scabies mite serpin expand the set of complement-inhibiting mite proteins, as these also prevent activation of all complement pathways in these ELISA-based functional assays. All SMIPP-Ss and SMSs investigated to date were previously localized by immunohistology [16], [24]. All are secreted into the mite gut and subsequently excreted as components of feces into the confined space of the mite burrows within the upper epidermal layers of the human skin. Taken together, the mite produces an astonishing repertoire of complement inhibitors to prevent complement activation within the mite gut and in its vicinity. While complement factors C1q and C9 are localized within the mite digestive system, the terminal complement MAC formation was not detectable in the mite gut, indicating that this anti-complement machinery may be very efficient *in vivo* [25]. By inference, such a situation favors secondary infections by bacterial pathogens and indeed, bacterial lawns are found to coat the mite burrows and gram-positive cocci have been isolated from mite fecal pellets [26].

We now argue that effective inactivation of complement by these scabies derived complement inhibitors may aid in efficient growth of GAS in the microenvironment of the burrows. We tested this in whole blood bactericidal assays employing functional human phagocytes and complement. We found that the presence of each of the four representatively chosen scabies proteins enhanced growth of GAS in these assays. Three individuals with normal activation levels of the two dominant complement pathways (CP and AP, Figure 2a), which were previously shown to be required for innate immunity to *S. pyogenes* [27], were recruited as human blood donors. Generally, mite complement inhibitors enhanced bacterial growth in GAS bactericidal assays using naïve donor blood, with or without added antibodies specific to the GAS strain used. However, the most striking effects of mite molecules were seen when strain specific antibodies were present. Growth of GAS in the presence of blood from donor D1, with a robust type-specific immune response to the GAS strain 2967, was significantly increased in the presence of SMIPP-S I1, SMSB3 and SMSB4. These results were similar with blood from donors (D2 and D3), who did not exhibit type-specific immunity to GAS 2967. A standardized assay, using naïve blood from donors D2 and D3 allowed testing of four mite proteins at a range of concentrations. These experiments showed dose dependent bacterial growth. Notably, the effects of some of the mite complement inhibitors tested in this system were most dramatic at concentrations below 100 µg/ml, which may be more relevant in a physiological context. Moreover, additive effects of multiple mite proteins accumulated in fecal pellets within the human skin burrows would be expected to cause strong local complement inhibition and may play an important role *in vivo*. Under physiological conditions the increase in bacterial survival may occur at relatively low concentrations of individual mite complement inhibitors.

This is the first molecular study suggesting a mechanism that may contribute to the positive association between scabies and GAS skin infection. We propose that the collective complement-inhibitory function of multiple scabies mite excretory proteins in combination with complement inhibitors produced by GAS [28] promote the survival of bacterial pathogens in the microenvironment of the epidermal burrows (Figure 3c). Their co-localization and our demonstration of their interactions clearly establish the potential worth of a concerted intervention against scabies in the control of secondary bacterial skin infections. These scabies mite proteins may present themselves as new targets for protective intervention at the onset of disease, both scabies and associated pyoderma.

As pyoderma is a condition caused by a combination of bacteria species, extending the ground-breaking studies presented here to further pathogens is imperative. Similar studies on *Staphyococcus aureus* are currently underway and show comparable preliminary data (not shown). Another important step will be to demonstrate the effect of mite molecules in an *in vivo* setting. Our group has developed a tractable experimental scabies porcine model [29]. We aim to study pyoderma development *in vivo* and to investigate the synergism between scabies mites and pathogenic bacteria in complement inhibition.

Our results strongly suggest that the misnomer “itch-mite” trivializes an important component of an increasingly urgent public health issue worldwide. More clinical emphasis should be given to the scabies component in controlling pyoderma in tropical settings. Understanding the biological relationship between host, mites and bacteria *in vivo* will promote the development of novel preventive and therapeutic strategies to control scabies and associated bacterial disease, likely translating into changes of policy and practice.
